# Study on the spatial-temporal variation in evapotranspiration in China from 1948 to 2018

**DOI:** 10.1038/s41598-020-74384-3

**Published:** 2020-10-13

**Authors:** Feng Zhang, Mengqing Geng, Qiulan Wu, Yong Liang

**Affiliations:** College of Information Science and Engineering, Shandong Agricultural University, Tai’an, 271018 China

**Keywords:** Ecology, Hydrology

## Abstract

It is of great significance for the efficient utilization of water resources and the construction of the ecological environment in China to fully understand the evolution process of the spatial-temporal pattern of evapotranspiration (ET). With the use of the v2.0 and v2.1 ET data sets combined with the Global Land Data Assimilation System and Noah model, this paper selects pixels as the basic research object to analyse the spatial-temporal variation in ET in China during the 71 years from 1948 to 2018. We first applied the TFPW-MK test to study the annual ET trend in China throughout the 71-year period, including the ET trend of each month from January to December and the annual total ET trend. Moreover, we examined the spatial variation in these trends. In addition, we calculated the variation coefficient of the time series of each pixel’s ET throughout the 71-year period and the variation coefficient of the spatial distribution of ET in each year to analyse the spatial-temporal fluctuations in ET in the study area. Finally, the Hurst index was adopted to evaluate the future ET trend. Based on these analyses, we observed the following novel spatial-temporal characteristics of ET: from 1948 to 2018, (1) the ET in most regions covered by 89.5% of all pixels in China exhibits an increasing trend. (2) The ET trend in China varies greatly with the change in months, and many regions show the most or least obvious increasing trend (or decreasing trend) at different times. (3) The area with an increasing trend is the largest in May and the smallest in December, and more than half of the pixels in all months of a year reveal an increasing trend. (4) In the northeast, west and south regions of China, the monthly fluctuation in the ET trend is relatively large, which indicates that the ET trend in these regions is greatly affected by the month. (5) The fluctuation in ET in China is larger in the north than it is in the south and larger in the west than it is in the east. The most stable fluctuation occurs in East China. (6) The ET trend of almost all the pixels in the study area remains consistent from 1948 to 2018, and there are large areas with a notable continuity. This results in the spatial variation in ET in the study area increasing.

## Introduction

Evapotranspiration (ET) is the total water vapor flux from vegetation and the ground to the atmosphere, including vegetation transpiration and soil evaporation^1^. ET is a comprehensive representation of land cover and water resources, a main form of the water cycle and an important part of water resources management^2–5^. ET plays an important role in global/regional climate models, hydrological cycle processes, agriculture, forestry and the environment^6^. The ET process reflects the exchange of material and energy. It is an important link in the dynamic balance of global ecosystems (atmosphere, hydrosphere, lithosphere and biosphere), the most important link connecting water cycle processes such as air-water, surface-water and groundwater cycle processes, and an important factor in the heat and water balances of water and land surfaces^7^. It is of great importance for the efficient utilization of water resources and the construction of the ecological environment in China to fully understand the evolution process of the spatial-temporal pattern of ET.

The traditional method of measuring and estimating ET through stations hardly obtains accurate data, which is often limited to the study of small areas and mostly focuses on certain area of increased interest and vulnerable areas, such as Northeast China^8–10^, the Loess Plateau^11,12^ and the Tibetan Plateau^13,14^. With the rise of remote sensing technology, the use of remote sensing data to estimate ET across a large area has become the main direction of current research^15,16^. In recent years, many experts and scholars have performed much research on the trend of ET in China, and their research results are very consistent. Based on NOAA-AVHRR remote sensing data, meteorological data, soil data and other auxiliary data from 1991 to 2000, using the improved ecosystem productivity simulator (BEPS), Zhou Lei et al.^17^ simulated the spatial distribution pattern of ET at different time scales in the Chinese terrestrial ecosystem, and analysed the temporal and spatial characteristics of ET in the Chinese terrestrial ecosystem and its response to climate change, their research results revealed that the ET in Chinese terrestrial ecosystems showed an upward trend in the 1990s. The highest value of ET over the 10 years occurred in 1998 when both the temperature and precipitation reached their peaks; the lowest value was observed in 1992, when the annual precipitation was the lowest. Bing Longfei et al.^7^ analysed and simulated the land ET and soil water content in China from 1986 to 2009 by the Noah land surface model according to six large areas and five ecosystem types. They examined the ET and soil water content variations in the different ecosystem types in China and in various regions and studied the relationship between the different types of ET and soil water content. Their research results indicated that since the 1980s, the land ET in China generally exhibited an increasing trend, and in the Chinese central-south, southwest, east, northeast, and northwestern regions, the ET trends were consistent with the general national trend, namely, an increasing trend, while the overall ET trend in North China revealed a declining trend. The period with the highest ET in North China was the 1990s. Tian Jing et al.^18^ simulated the changes in surface water and heat process parameters in mainland China from 1986 to 1996 and from 2000 to 2008 based on the Noah land surface process model. According to the six major regions of mainland China (Northeast China, North China, East China, Central South China, Northwest China and Southwest China), the spatial-temporal characteristics of the surface ET in these six regions since 1986 were analysed, and their research results showed that since 1986, except for the decline in the annual ET in North China, the annual ET in the remaining Northeast, East, Central South, Northwest and Southwest regions increased, i.e., the overall trend was an increasing trend. The above research results on the change trend of the Chinese ET are basically the same, namely, since the 1980s, the Chinese ET has generally increased, while the ET in North China has generally decreased. However, these studies were based on predefined regions, such as specific land cover types, administrative regions or certain ecological regions, and statistical and comparative analyses were performed of the average or total ET in the above defined areas in time and space dimensions. The regions defined by other factors are relatively large, which does not reflect the distribution characteristics of ET across the internal space of the area. The above studies did not consider the heterogeneity of the ET in the predefined regions, which may mask certain characteristics of the spatial-temporal variation in ET in China, and the research time scale was relatively short.

Xiangyi Li et al.^19^ developed a high-resolution data-oriented monthly ET product for China between 1982 and 2015 by integrating remote sensing and the eddy covariance technique to observe ET data in a machine learning approach (model tree ensemble, MTE). Based on these data, they found certain characteristics of the spatial-temporal patterns of ET in China. However, the focus of the above study was to develop data products using machine learning methods and to compare and analyse these data products with other data products without a deep and detailed analysis of the spatial-temporal variation in ET in China. For the trend study, the researchers chose the ET in certain years over the study period to perform subtraction operations to determine the trend. They did not consider the annual change in ET over the study period.

Although these studies have examined many spatial-temporal characteristics of the ET in China, certain problems remain to be further investigated: (1) although the past ET trend in China has been studied based on long time series, the spatial variation in this trend requires further assessment. (2) The temporal variation in the annual spatial variation in ET in China and the spatial variation in ET over a long time series need to be studied. (3) The possible future ET trend in China should be studied.

In response to the above problems, in contrast to the current ET research in China, we studied the spatial-temporal variation in ET from a new perspective. This paper emphasized the change process of ET, assessed the spatial-temporal characteristics of the ET trend and determined the spatial-temporal variation characteristics and future trend of ET in detail to mitigate the above shortcomings of current research, and certain spatial-temporal variation characteristics of ET were identified that have not been reported before. The contributions of this paper are as follows: (1) With the use of the TFPW-MK test method, this paper studied the annual variation in ET trends from year-round and monthly perspectives in China throughout the study period, and the spatial variation in these trends was also studied. (2) The variation coefficient of the time series of each pixel’s ET throughout the study period and the variation coefficient of the spatial distribution of ET were calculated to analyse the spatial-temporal fluctuation in ET in China. (3) This paper analysed the possible future trend of the ET in China by the Hurst index. (4) This paper extended the study period to 71 years from 1948 to 2018.

The structure of this paper is as follows: the second section describes the data source and the details of the data statistical methods adopted in this study; the third section analyses the results of the data statistics and attempts to determine the spatial-temporal characteristics of ET; the last section draws the conclusion of the study and summarizes this paper.

## Data and methods

### Data source

The data used in this paper originate from the Global Land Data Assimilation System (GLDAS) of the European Space Agency (ESA). The GLDAS is a data assimilation system based on satellites, land surface models and ground observations. It provides land surface data from 1948 to now, at a spatial resolution of $0.25^\circ \times 0.25^\circ$ and $1^\circ \times 1^\circ$, and a temporal resolution of 3 hours and 1 month^20^. The meteorological driving data used in the GLDAS come from multisource observations, reanalysis data and atmospheric assimilation products, which attain a high applicability in regional research. Jiang et al.^21^ and Wang Wen et al.^22^ confirmed the applicability of the GLDAS in China, and the data have been widely applied in the study of climate and water resources in China^23–27^, and in other parts of the world^5,28,29^. In this paper, we adopt the v2.0 and v2.1 ET data sets combined with the GLDAS and Noah model to evaluate the spatial-temporal variation in ET in China. The v2.0 data period spans from 1948 to 1999, and the v2.1 data period spans from 2000 to 2018. There is no v2.0 data set for the period from 1948 to 1999 All the data have a spatial resolution of $0.25^\circ \times 0.25^\circ$.

The research area of this paper is mainland China and two islands in the southeast of China. We did not consider the ET on the other smaller islands in the South China Sea, and these islands in the South China Sea are not shown on the map in this paper.

### Trend free prewhitening Mann-Kendall trend test

The Mann-Kendall (M-K) test method is recommended by the World Meteorological Organization and has been widely applied^30^, because it has the advantages of not being affected by sample values, distribution types, etc. This test method is very effective for trend analysis of precipitation and other natural event time series. This method has been adopted to test the ET trend and verify its applicability^31–33^.

However, with the deepening of research, certain shortcomings of this method have also been exposed. V. Storch^34^ stated that the autocorrelation of the original data could lead to the enhancement or decrease in the detection results to a certain extent, so the prewhitening MK (PW-MK )test method was proposed. Zhang Xuebin et al.^35^ and D.H.Burn et al.^36^ proposed that preset white processing reduced the MK test ability to detect significant results. Sarr et al.^37^ applied both visual examination and a modified version of the Mann-Kendall (MM-K) test to assess the trends of the time series of averaged indices. Sheng Yue et al.^38,39^ found that for an autocorrelated sequence with trend terms, certain trend terms were removed by preset white processing, leading to the acceptance of an invalid hypothesis, and the TFPW-MK (trend-free prewhitening Mann–Kendall) test method was proposed. The TFPW-MK method has been applied in many studies, and good results have been achieved^40–42^.

For time series variables $X_t(t=1, 2 \ldots,n)$, where *t* is the length of the time series, the specific steps of the TFPW-MK method are as follows:

*Step 1*. Calculate the inclination $\beta$ of the time series variables.

$$\begin{aligned} \beta = median \left( \frac{x_j-x_i}{j-i}\right) , \quad \forall j>i \end{aligned}$$

*Step 2*. Remove any trend items from the time series variables to form a sequence without trend items.

$$\begin{aligned} Y_t = x_t - \beta t, \end{aligned}$$

*Step 3*. Calculate the first-order coefficient of autocorrelation.

$$\begin{aligned} r_1 = \frac{\sum \limits _{t=1}^{n-1}(x_t - \overline{x_t})(x_{t+1} - \overline{x_{t+1}})}{\sqrt{\sum \limits _{t=1}^{n-1}(x_t - \overline{x_t})^{2}\sum \limits _{t=1}^{n-1}(x_{t+1} - \overline{x_{t+1}})^{2}}}, \end{aligned}$$

*Step 4*. Perform a significance test on $r_1$.

$$\begin{aligned} r_\alpha (\alpha = 0.1) = \frac{1 \pm 1.645 \sqrt{n-2}}{n-1}, \end{aligned}$$

When $|r_1|> r_\alpha$, $Y_t$ passes the significance test and proceed to steps 5, 6 and 7. If $Y_t$ fails the significance test, perform the seventh step of the M-K test directly on the $Y_t$ sequence.

*Step 5*. Eliminate the autocorrelation items.

$$\begin{aligned} Y'_t = Y_t - r_1Y_{t-1}, \end{aligned}$$

*Step 6*. Supplement the trend term $\beta t$ to obtain a new sequence without an autocorrelation effect.

$$\begin{aligned} Y''_t = Y'_t + \beta t, \end{aligned}$$

*Step 7*. Perform the M-K Trend test on the new data series $Y''_t$.

The M-K method defines the following statistics:

$$\begin{aligned} S=\sum \limits _{k=1}^{n-1}\sum \limits _{j=k+1}^{n}sgn(x_j-x_k) ~~~~~~~~j,k=1,2,...,n \end{aligned}$$

where, *n* is the total number of samples and $x_j$ and $x_k$ are sample values at times *j* and *k* respectively. *sgn*() is a symbolic function with the following rules:

$$\begin{aligned} sgn(x_j-x_k)= \left\{ \begin{aligned} 1,\quad x_j-x_k>0 \\ 0,\quad x_j-x_k=0 \\ -1,\quad x_j-x_k<0 \\ \end{aligned} \right. \end{aligned}$$

*S* is a normal distribution with a mean value of 0 and a variance of $var(S)=n(n-1)(2n+5)/18$. When $n >10$, the normal distribution statistics are calculated as follows:

$$\begin{aligned} Z= \left\{ \begin{aligned} \frac{S-1}{\sqrt{var(S)}},~~~~S>0 \\ 0, ~~~~S=0 \\ \frac{S+1}{\sqrt{var(S)}},~~~~S<0 \\ \end{aligned} \right. \end{aligned}$$

If $Z > 0$, this indicates that ET exhibits an increasing trend over this period; otherwise, it exhibits a decreasing trend, and the larger the absolute value is, the more obvious the trend is. When the absolute value of *Z* is larger than or equal to 1.28, 1.64 and 2.32, the confidence levels are 90%, 95% and 99%^43,44^, respectively.

In terms of the data used in this paper, the ET values of the pixels at certain locations from 1948 to 2018 constitute a time series. Then the *Z* value of ET can be calculated by this time series.

### Coefficient of variation

The coefficient of variation (CV) is a statistic that measures the dispersion of each observation value, which is the ratio of the standard deviation to the mean. In this paper, the CV is adopted to characterize the dispersion of the ET time series or the spatial distribution of each pixel from 1948 to 2018. In regard to the time series data, the larger the CV is, the larger the data fluctuation is, and the time series data are unstable; conversely, the more centralized the time series data distribution is, and the time series data are stable. Regarding the spatial distribution of the data, the significance of the CV is similar. For the time series used in this paper, the calculation method of the CV is as follows:

$$\begin{aligned} C_v= \frac{\sigma }{{\overline{EVAP}}}, \end{aligned}$$

where $C_v$ is the CV, $\sigma$ is the standard deviation of the ET time series data, and ${\overline{EVAP}}$ is the average value of the ET time series data.

### Hurst index analysis

The Hurst index^45^ can predict the trend of future data based on long time series data, and it is an effective method to quantitatively describe the long-term dependence of time series information^46^. It has been widely applied in meteorology, hydrology and other fields^47,48^. The specific calculation process is as follows:

The ET time series is defined as $EVAP_{(t)}$, where $t=1,2,3,...,n$. For any positive integer $\tau \ge 1$, the mean value sequence of the time series is:

$$\begin{aligned} \overline{EVAP_{(\tau )}}= \frac{1}{\tau }\sum \limits _{t=1}^{\tau }EVAP_{(t)}, ~~~~~~~~\tau =1,2,...,n \end{aligned}$$

The cumulative dispersion sequence is:

$$\begin{aligned} X_{(t,\tau )}= \sum \limits _{t=1}^{\tau }(EVAP_{(t)}-\overline{EVAP_{(\tau )}}) \end{aligned}$$

The range sequence is:

$$\begin{aligned} R_{(\tau )}= maxX_{(t,\tau )}-minX_{(\tau )}~~~~~~~~1\le t \le \tau \end{aligned}$$

The standard deviation sequence is:

$$\begin{aligned} S_{(\tau )}= \left[ \frac{1}{\tau }\sum \limits _{t=1}^{\tau }(EVAP_{(t)}-\overline{EVAP_{(\tau )}})^2 \right] ^{\frac{1}{2}} \end{aligned}$$

The Hurst index is calculated as:

$$\begin{aligned} \frac{R_{\tau }}{S_{\tau }}=(c \tau )^H, \end{aligned}$$

where *H* is the Hurst index and *c* is a proportional parameter. Compute the logarithm of both sides of the above equation, apply the least square method to fit the data in the double logarithm coordinate system (ln$(R_{\tau }/S_{\tau })$, ln${\tau })$, and determine the slope of the straight line as the Hurst index. The value range of the Hurst index is (0,1), which can be divided into three cases:

1. When $0< H < 0.5$, the sequence exhibits reverse persistence, namely, the future trend is the opposite to the past trend, and the closer H is to 0, the stronger the reverse persistence is;
2. When $H = 0.5$, the sequence is a random sequence, namely, the future trend is independent of the past trend;
3. When $0.5< H < 1$, this indicates that the future trend is consistent with the past trend, and the closer H is to 1, the higher the sustainability is.

## Results

### Trend analysis of the ET from 1948 to 2018

To reveal the ET trend in the 71 years from 1948 to 2018 in the study area, we extracted the ET in the study area throughout this period from the GLDAS data, calculated the *Z* value of each pixel throughout this period with the TFPW-MK test method, and generated an ET change trend distribution map with the *Z* value of each pixel.

First, we adopt the annual ET of each pixel as the statistical value to establish the 71-year time series. The trend of each pixel from 1948 to 2018 is analysed to examine the general trend of the ET in China and its spatial distribution characteristics. Then, we select the ET of each pixel in each month from January to December as the statistical value, establish 12-month time series over the 71-year study period, and analyse the trend in each month over the 71 years from January to December to examine the influence of the month on the ET change trend in China.

#### Trend analysis of the ET over the years

**Figure 1:**
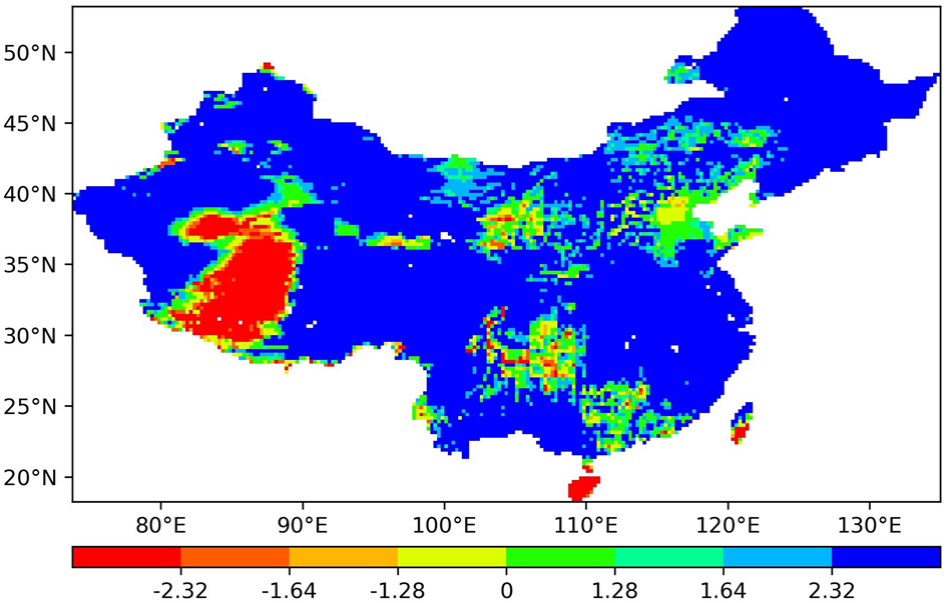
Spatial-temporal trend of the ET in China from 1948 to 2018.

First, we analyse the annual ET trend of each pixel throughout this period, and Fig. 1 shows the distribution of the Z value reflecting this trend.

According to the obtained statistics, there are approximately 15258 pixels in the study area, of which 13662 pixels exhibit Z values larger than 0, accounting for approximately 89.5% of all pixels. The other pixels with Z values smaller than 0 account for approximately 10.5% of all pixels. This shows that the overall trend in most regions of China since 1948 is an increasing trend, and only a small part exhibits a decreasing trend. Figure 1 shows that the regions where ET has significantly decreased are distributed across parts of Western China and the two islands in southern China, while the ET in most other regions exhibits a relatively significant growth trend.

Figure 1 shows that the ET change trend in Western China is quite different. The change trends in most areas are consistent with the overall ET change trend in China, showing a significant upward trend. The ET in a small part of the area (the red area in the figure), namely, the western Qiangtang Plateau and its surrounding areas, exhibits a significant downward trend. The Qiangtang Plateau is the main body of the Qinghai-Tibet Plateau in southwestern China. Most of the plateau is above 4600 metres above sea level. It is a typical area with very harsh climate conditions and an extremely fragile ecological environment in China. The environmental characteristics are mainly exemplified by a dry and cold climate, windy conditions, and abundant surface sand areas, sparse vegetation and a low ecological capacity^49^. Since the 1950s, the western Qiangtang Plateau has increasingly become arid with global changes^50^, and the precipitation in the southern surrounding area has decreased significantly^51^.These factors together led to the most obvious ET decreasing trend on the western Qiangtang Plateau and its surrounding areas in Southwest China. The reason for the significant increase in ET in Western China is basically the same as the reason for the increase in ET in the other parts of China, namely, climate change and human activities. Climate change is mainly due to the increase in precipitation^17^ and the increase in warming and aridification in most parts of China, which has greatly increased the temperature and relative humidity^52^. However, the increase in human activities is primarily caused by the fact that since 2000, the state has heavily invested in ecological restoration and has successively implemented a number of major ecological environmental protection and construction projects, such as returning farmland to forestland and grassland, returning grazing land to grassland, natural forest protection, and forest system protection projects. With the implementation of the above ecological projects, the vegetation conditions in certain areas have been improved^53^, and the areas where the ET has notably increased are mainly located in areas with a high vegetation cover^54^.

When the absolute *Z* value is greater than or equal to 2.32, the confidence level is 99%, and when it is greater than 1.64 but less than 2.32, the confidence level is 95%. When the absolute *Z* value is greater than 1.28 but less than 1.64, the confidence level is 90%. Table 1 lists the proportion of the number of pixels in each distribution interval of the *Z* value. The *Z* value in 63% of all pixels is greater than 2.32, and the areas covered by these pixels have a 99% chance of exhibiting an increasing trend. Analogously, the areas with *Z* values greater than 1.28, accounting for 89.7% of all pixels, have a 90% chance of exhibiting an increasing trend. All of these statistics indicate that in China, the ET in most regions exhibits a very obvious increasing trend.

**Table 1:** Confidence level of the *Z* value and pixel proportion.

*Z* value range	Confidence level (%)	Percentage of pixels (%)
$\le -2.32$	99	4.3
− 2.32 to − 1.64	95	1.6
1.64 to − 1.28	90	0.9
− 1.28 to 0	< 90	4.3
0–1.28	< 90	9.1
1.28–1.64	90	4.1
1.64–2.32	95	9.5
≥ 2.32	99	66.1

#### Variation trend of ET with the different months

**Figure 2:**
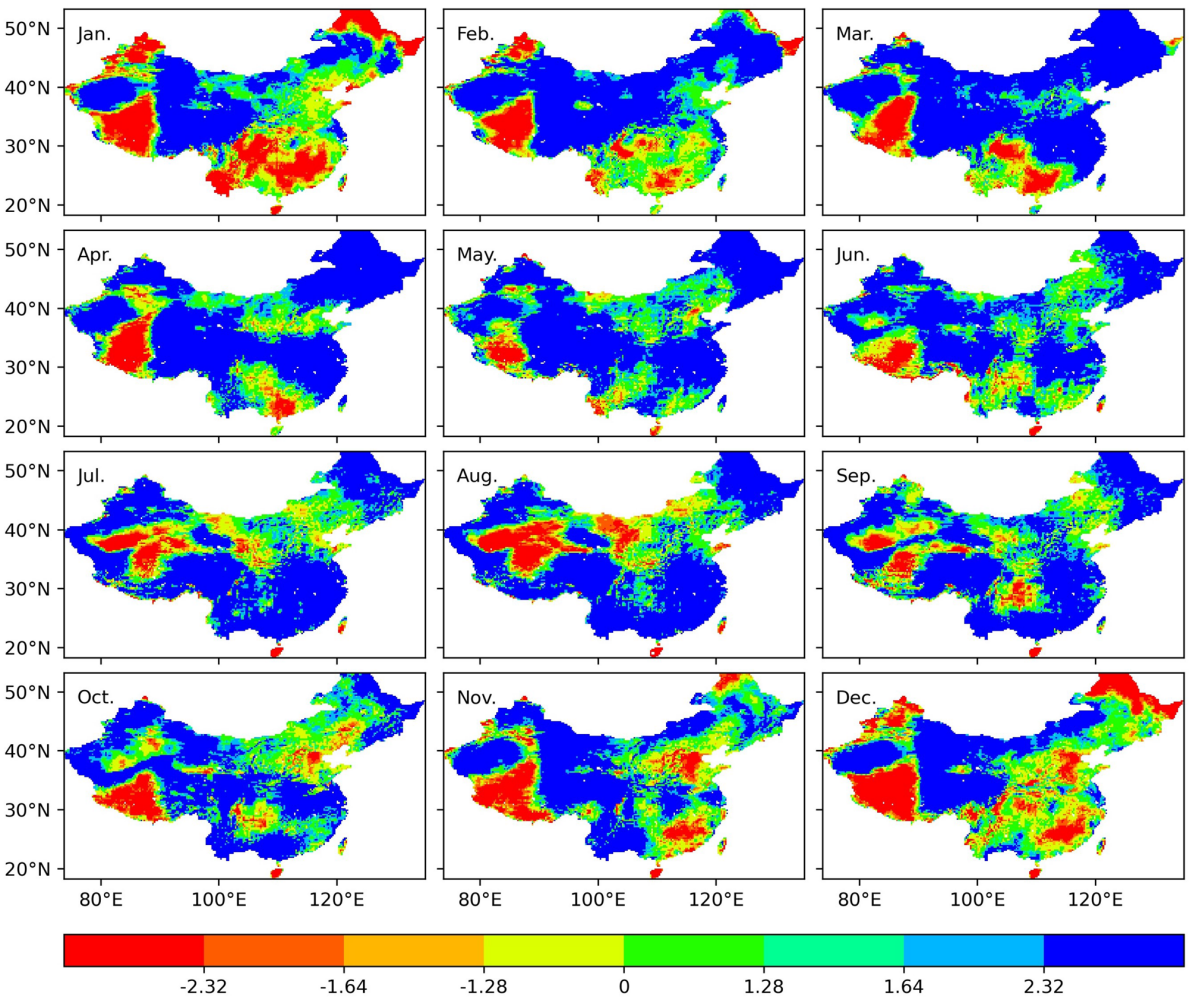
Spatial-temporal trend of the monthly ET in China from 1948 to 2018.

From January to December, solar radiation changes with the time, temperature, precipitation and other meteorological elements, and ET also changes over time^55^. To reveal the ET trend with the month, we calculated the ET trend of each pixel throughout the 71-year period from January to December. Figure 2 shows a distribution map of the Z value reflecting this trend.

Figure 2 reveals that the ET trend in China varies greatly with the change in months, and many regions show the most or least obvious increasing trend (or decreasing trend) at different times. The details are as follows:

1. In Northeast China, especially the Middle-Lower Yangtze Plain and the eastern Tibetan Plateau, the ET increasing trend is the most obvious in April and the least obvious in January and December.
2. On the North China Plain, the ET increasing trend is the most obvious in March, and the ET decreasing trend is the most obvious in November and December.
3. On the Yunnan-Guizhou Plateau and Chiang-nan Hilly Region, ET increased the most from June to August and decreased the most in January.
4. The increasing trend on the Inner Mongolia Plateau is the most obvious in February and March and the least obvious in August.
5. Compared to the other months, the increasing trend on the western Tibetan Plateau from May to September is more obvious. However, the annual ET increasing trend is not obvious, but the decreasing trend is very obvious.
6. The decreasing trend in January and December in Northwest China is obvious, and the increasing trend in the other months is obvious.
7. The annual ET trend in the Tarim Basin and its surrounding areas is an obvious increasing trend. However, the ET trends in the east and west of the Tarim Basin are obviously different. In August and September, the west of the Tarim Basin reaches the maximum value of the ET trend, while the east of the Tarim Basin exhibits the most obvious decreasing trend.

In general, the ET trend in Northeast China varies greatly from month to month. The ET in most areas of Northeast China mainly increases from March to October, while the ET mainly decreases from December to February. This is related to the concentrated distribution of the forest areas in the Greater Khingan Mountains and Changbai Mountains in Northeast China. The ET in forest ecosystems is the highest. From March to October every year, plants are subject to the growing season, the vegetation is lush, transpiration and evaporation occur vigorously, and ET is on the rise. From December to February of the following year, plants are in the declining or non-growing season. Moreover, due to the low temperature, energy and stomatal conductance levels, the ET values reveal a downward trend^54^.

The variation trend of the ET in southern China, northwestern China, and northern China is also relatively obvious. The ET from June to August mainly reveals an upward trend, and the ET mainly shows a downward trend from September to May of the following year. This is mainly related to the temperature and precipitation. Between June and August, the temperature and precipitation increase, and the ET is also very notable; from September to May of the following year, the temperature drops, and the precipitation and ET also decrease^56^.

However, the ET in most parts of Southwest China exhibits a downward trend in almost all months, but it is also observed that the area with a decreasing trend in winter is relatively large, while that in summer is relatively small. The main reason why the ET in each month decreases is that this region is located on the Qiangtang Plateau, an alpine and cold region with an altitude higher than 4600 metres, which has a unique natural environment and climatic conditions^51^. Drought and precipitation reduction are the leading factors of the ET decrease^50,51^, , and the ET throughout the whole year mostly presents a downward trend. Moreover, the monthly changes in the climate of the Qiangtang Plateau are very obvious, with distinct cold and wet seasons. Generally, the period from May to September is the warm, rainy and less windy season, but the period from October to April of the following year is the cold, dry, and windy season^50^, and the area with an ET decreasing trend from May to September shrinks, while the area with an ET decreasing trend from October to April expands.

**Figure 3:**
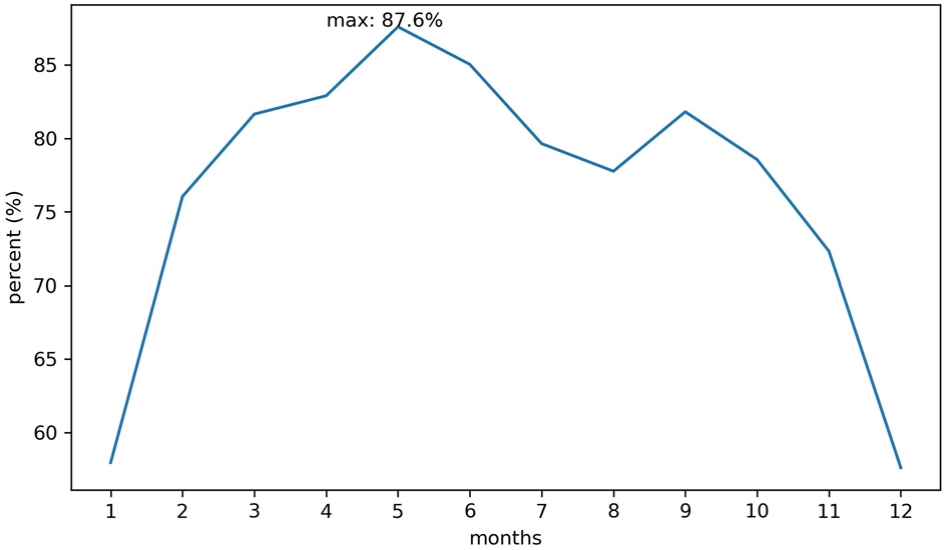
Proportion of pixels with an increasing trend over the 12 months.

According to the monthly change trend, we calculated the proportion of the number of pixels with an increasing trend, i.e., those pixels with Z values greater than 0. Figure 3 shows the calculation results. The figure shows the ET trend in China with the change in months.

Figure 3 shows that in January, only 57.95% of the study area exhibited an increasing trend, which quickly increased to over 76.0% in February, reaching a maximum value in May, after which it began to decrease. However, the area increased in September, rising to 81.81%, and then continued to decrease, until it reached a minimum value in December, similar to January. On the whole, the proportion of $Z>0$ from January to December was $>50\%$, and all months of the year were dominated by a growth trend, and from January to May, the pixels with $Z>0$ increased, with a total increase of 29.65%. The growth rate was the highest from January to February, with a total increase of 18.05%, accounting for 60.88% of the increase, indicating that the area where the ET was on the rise from January to February exhibited the fastest growth, and the number of pixels with $Z>0$ reached a maximum value of 87.60% in May, after which it decreased. There was a small fluctuation in the middle of September, but an overall decrease was still observed, and the rate of decrease increased, with a total decrease of 30%, reaching a minimum value of 57.60% in December, which was still higher than 50%. From Fig. 3,we can deduce that the number of pixels with an increasing trend in the study area was the largest in May and the smallest in December and January. In particular, the region in the study area with an increasing trend was the largest in May and the smallest in December. In all months of the year, more than half of the pixels exhibited an increasing trend, which also indicates that in regard to the study area, the annual ET trend was still dominated by an increasing trend, which is consistent with the finding from Fig. 1.

**Figure 4:**
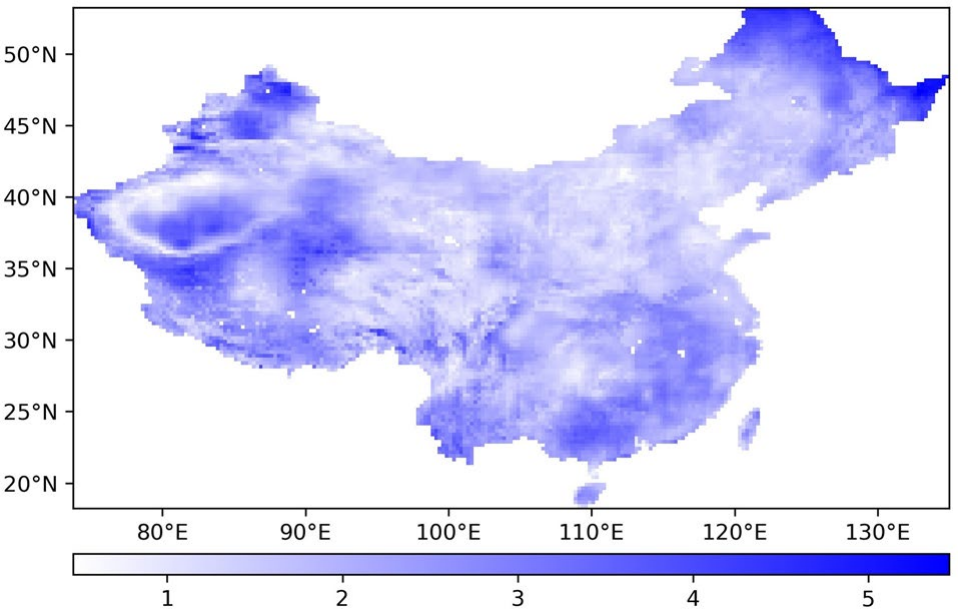
Standard deviation distribution of the monthly ET trend in China.

Figure 2 does not directly show the monthly fluctuation in the ET trend of each pixel from January to December. Standard deviation analysis of the 12 subgraphs of Fig. 2 is conducted, and Fig. 4 is obtained. The standard deviation can be adopted to analyse the dispersion of the Z value of each pixel from January to December, and based on Fig. 4 we can determine the monthly fluctuation in the ET trend.

Figure 4 shows that in the dark blue parts, i.e., in Northeast China, West China, Northwest China and South China , a large variation occurs, especially in the border area of Northeast China and a small number of pixels in Northwest China, where the standard deviation exceeds 4.5, indicating that the ET trend in these regions is greatly affected by the months. In regard to the light blue parts of the map, such as the northwest Tarim Basin, Tianshan Mountains and its surrounding areas, east Tibet Plateau and middle Inner Mongolia Plateau, the impact of the month is relatively small.

### Coefficient of variation analysis

Through statistical analysis of the ET CV in time and space, the dispersion of ET in time and space can be analysed, and the stability of the ET fluctuation in time and space can then be determined.

#### Spatial distribution of the time series CV of ET

**Figure 5:**
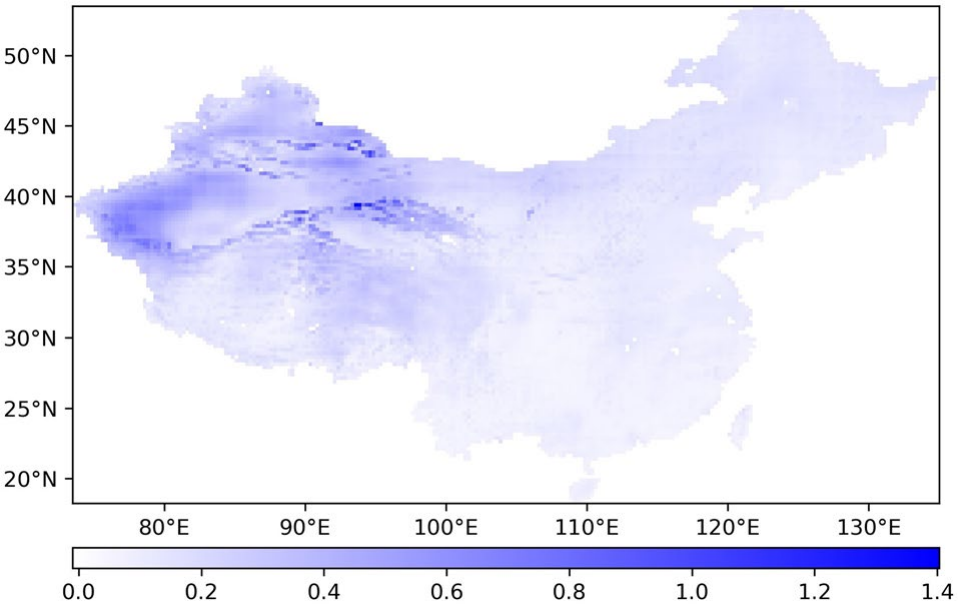
Spatial distribution of the time series CV of ET.

The time series CV of ET from 1948 to 2018 is calculated for each pixel, and Fig. 5 is obtained.

Figure 5 shows that the ET CV of each pixel in China from 1948 to 2018 shows a trend of gradually decreasing from northwest to southeast. The ET in northern China is more discrete than that in the south, and the ET in the west is more discrete than that in the east. The higher the dispersion degree is, the more unstable the ET in these regions is over the 71-year period. The lower the dispersion degree is, the more stable the change in ET is.

In summary, from 1948 to 2018, the variation in ET in northern China was more severe than that in southern China, and the variation in ET in Western China was more severe than that in eastern China. The ET in the surrounding areas of the Tarim Basin in northwestern China revealed the most dramatic changes, and the ET changes in most parts of East China remained the most stable.

#### Time fluctuation in the spatial distribution CV of ET

**Figure 6:**
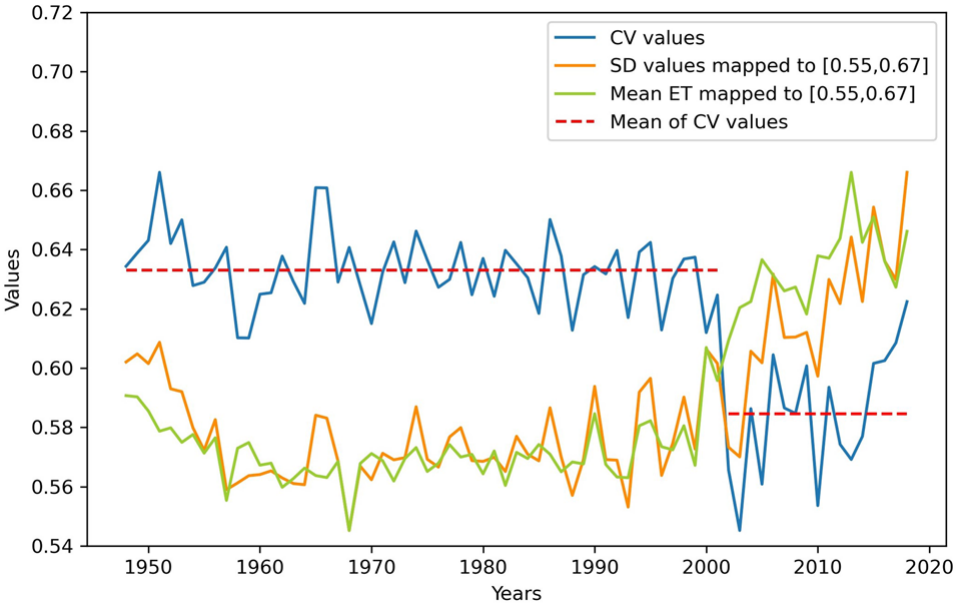
Time fluctuation in the spatial distribution CV of ET.

From 1948 to 2018, the CV of the yearly ET spatial distribution in the study area was calculated to analyse the fluctuation in the ET spatial variation over time. Figure 6 shows a linear graph based on the 71 CV yearly values, from which we can observe the changes in the spatial variation from 1948 to 2018. Moreover, we also calculated the SD and mean from 1948 to 2018. To facilitate a comparison of the change trends of the SD and mean with the change trend of the CV, we mapped the SD and mean to the range of the CV, [0.55, 0.67], and accordingly plotted a line graph of the SD and mean.

In Fig. 6, the yellow solid line is the change curve of the SD over time, and the blue solid line is the variation line of the CV over time. The blue solid line reveals that the change in the spatial distribution CV over time from 1948 to 2018 can be roughly divided into two stages: 1948–2001 and 2002–2018. The red dotted line is the mean value of the CV in each year in the two stages. The following is a description of these two stages:

The first stage: 1948–2001. During this period, the CV value of ET fluctuated within a high range, ranging from 0.61 to 0.67, and the average value was approximately 0.63, but the fluctuation range in most years was approximately 0.62 to 0.65, and the change was relatively small. Among them, the CV of ET in 1959 and 2000 was relatively small, indicating that the ET in the study area in these two years remained relatively uniform, while the CV in the other years (such as 1951, 1965, and 1986) were relatively large, indicating that the ET in the study area varied greatly in these years. However, on the whole, the CV in each year in this stage was larger than that in the second stage, indicating that the spatial difference in ET in the study area in this stage was large, and the overall ET was uneven.

The second stage: 2002–2018. Figure 6 shows that the CV began to decrease in 2002, and it decreased to a minimum value of 0.55 in 2003. Thereafter, up to 2018, the value of the CV fluctuated within a low range, ranging from 0.55 to 0.62, with an average value of 0.58, which is a decrease of 0.05 over the first stage value. Although it reached a maximum value of 0.62 in the second stage in 2018, the value was smaller than the average value in the first stage , indicating that the CV in this stage was generally smaller than that in the first stage. Notably, the difference in ET between the various regions in the study area decreased in 2002, and the ET in China became more even. According to the change curve of the average ET, the average ET in China began to increase in 2002. Although fluctuations occurred, the average ET also fluctuated within a relatively high range. This is consistent with the research results of Bing Longfei^7^, namely, after 2000, the annual ET greatly exceeded the previous ET level. Combined with the decrease in the CV value, this shows that after 2002, the ET in the various regions of China started to increase. The main reason for this result is that the state invested heavily in ecological restoration in 2000 and successively implemented a number of major ecological environmental protection and construction projects, such as returning farmland to forestland and grassland, returning grazing land to grassland, natural forest protection and shelterbelt system projects^53^. After 2002, good results were achieved, and vegetation conditions were improved, while the regions with a notably increased ET primarily occurred in those regions with an improved vegetation cover^54^. Therefore, after 2002, the ET in all regions in China began to increase, and the CV began to decrease.

By comparing the SD line chart and the CV line chart, it is observed that the trend of these two lines in the first stage and the second stage is basically the same, but after the first stage, the CV exhibits a decrease, the SD does not change, and there is an increasing trend after 2002. However, the SD is an absolute indicator. When the sample mean level is different, an absolute difference index cannot be considered in a comparative analysis^57^, while the CV measures the degree of variation between samples with different units or with a large difference in the mean. Here, the annual average ET value constantly changes, and it is more accurate to adopt the CV to compare the dispersion degree between the different regions within the study area.

In other words, the annual ET spatial difference within the study area was relatively large from 1948 to 2001, and the annual ET in the study area was very uneven. After 2002, the annual spatial difference decreased, and in 2003, the spatial distribution of the ET in the study area was the most uniform.

### Future trend analysis of ET

**Figure 7:**
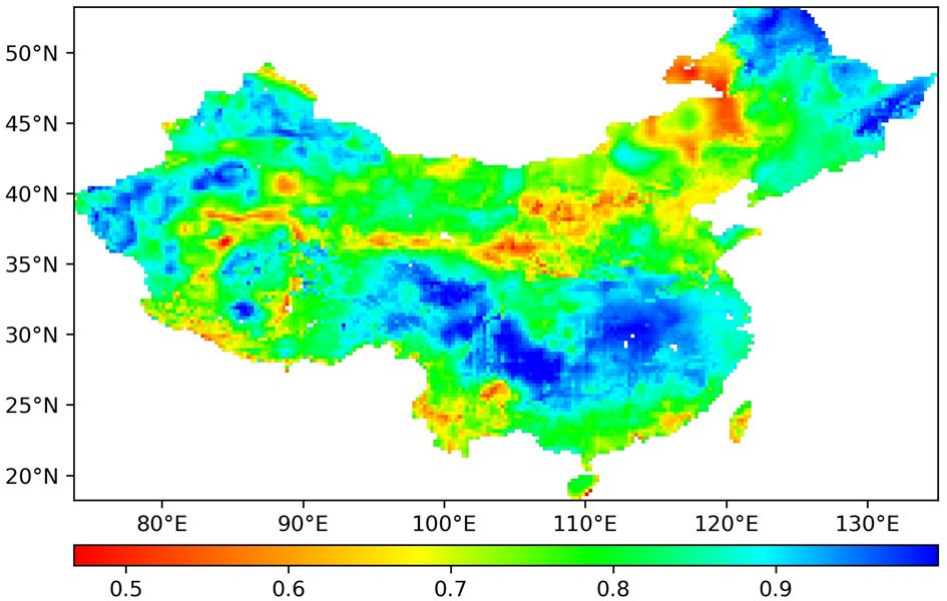
Spatial distribution of the Hurst index from 1948 to 2018.

The variation in ET in the study area from 1948 to 2018 has been previously analysed. This section assesses the future ET variation in China, i.e., whether the future ET variation in the study area will follow the trend from 1948 to 2018. This is evaluated with the Hurst index. The value range of the Hurst index is between 0 and 1. If the Hurst index is larger than 0.5, this indicates that the future trend will follow the original trend. The closer the Hurst index is to 1, the stronger the continuity is. If the Hurst index is smaller than 0.5, this indicates that the future trend will contradict the original trend. If the Hurst index is equal to 0.5, this indicates that the future trend is uncertain and not related to the original trend. Figure 7 shows a map of the distribution based on the calculated Hurst index of each pixel.

According to the calculated Hurst index of each pixel, only 26 pixels in Fig. 7 have a Hurst index smaller than 0.5, no pixels exhibit a Hurst index equal to 0.5, and most of the pixels in the study area reveal a Hurst index larger than 0.5. This implies that in the future, the vast majority of the study area will continue the trend from 1948 to 2018, as shown in Fig. 1. In terms of the possibility of this continuity, the number of pixels with a Hurst index larger than 0.9 accounts for approximately 23.2% of all pixels, and the number of pixels with a Hurst index between 0.8 and 0.9 accounts for approximately 36.8% of all pixels, while the number of pixels with a Hurst larger than 0.8 accounts for approximately 60% of all pixels. These results indicate that it is very possible for these pixels to continue the current trend in the future. Especially in Northeast China, South-Central China and West China, the Hurst index values are all close to 1, and the ET trend in these regions exhibits a notable continuity. For example, according to Fig. 1, it is found that the ET in Northeast China has a strong increasing trend from 1948 to 2018. Combined with the Hurst index analysis results in Northeast China, as shown in Fig. 7, it is concluded that in the future, the ET in Northeast China will increase more than that in the other regions.

## Conclusion and discussion

### Conclusion

The research results of this paper are basically consistent with previously published relevant research results. For example, this study is consistent with that of He Tian et al.^54^ whereby the areas with an increasing ET mainly occur on the Huang-Huai-Hai Plain and the Northeast Plain, while the areas with a significant decreasing trend are primarily distributed on the central Qinghai-Tibet Plateau, central Inner Mongolia and northern Xinjiang. Our results are consistent with the research results of Bing Longfei, Tian Jing and Zhou Lei indicating that the Chinese ET has generally been increasing since the 1980s, and the ET in North China has been decreasing.

In addition, in this study, the following conclusions on the temporal and spatial variabilities in the Chinese ET are obtained:

1. The ET in most parts of China is increasing in all months. The region with a growth trend in the study area is the largest in May and the smallest in December.
2. The change trend of the ET in China varies greatly with increasing month in the different regions. With increasing month, the ET in the northeastern, western and southern regions of the study area fluctuates greatly, especially along the borders of the northeastern region. A small number of pixels in the nearby and northwestern regions exhibit the largest monthly fluctuations, which indicates that the ET trends in these areas are more affected by the monthly changes. In most of the central part of the study area, the ET change trend is less affected by the monthly changes and primarily is an increasing trend.
3. From 1948 to 2018, the fluctuation in the ET in the study area is greater in the northern region than that in the southern region and is greater in the western region than that in the eastern region. The ET fluctuations in most of the eastern region remain the most stable, and the ET in the surrounding areas of the Tarim Basin in the northwest exhibit the largest fluctuations.
4. From 1948 to 2001, the annual ET in the study area is highly dispersed, and the ET in each area is very uneven. From 2002 to 2018, the difference in ET among the various regions in the study area is small, and the ET in the study area becomes more uniform. Notably, the spatial difference in ET in 2003 is the smallest.
5. In the future, the ET change trend in almost all pixels in the study area will continue that from 1948 to 2018, and there is a strong continuity in a large part of the study area. This continuity includes both the continuity whereby the ET continuously increases in Northeast China and the continuity whereby the ET continuously decreases in Southwest China .

In summary, from 1948 to 2018, the ET in most areas in the study area exhibits an increasing trend, and a few areas exhibit a decreasing trend. The spatial difference in ET is continuously increasing, and the future ET changes in these areas will follow the current trend. Therefore, the spatial difference in ET between the different parts of the study area tends to increase over time, the variability in the monthly ET has become more obvious, and the variability in ET in time and space has become more serious. Moreover, the research data analysed in this study have been proven to exhibit a high applicability to other parts of the world^5,28,29^. Relevant research methods have also proven their applicability in studying long-term natural events, which have been applied in other parts worldwide^58,59^. Therefore, in other regions with similar or different climates, relevant research data and research methods could be adopted to analyse the results.

### Discussion

#### The uncertainty of this research

The uncertainty of this research mainly originates from the following aspects. At present, the parameterization scheme in the remote sensing ET model is relatively simple, and there is an insufficient understanding of the spatial heterogeneity in the water and heat transfer of the underlying surface and the interaction mechanism of the soil-vegetation-atmosphere system. The estimation of ET by remote sensing involves many control factors of complex surface processes (climate, soil properties and plant biophysics, etc.), and the mismatch between the remote sensing inversion data and model input data in addition to the scale also generate uncertainties, resulting in a lower measurement accuracy of remote sensing means than that of traditional site observations^56^. The spatial resolution of the Noah model simulation results is $0.25^\circ$. When large-scale analysis is performed, although the overall trend and law are maintained, this will still cause uncertainties^18^.

#### Future research

Future research should mainly be conducted in the following aspects:

1. Perform a more reasonable calibration of the parameters introduced in the remote sensing ET model and conduct a detailed verification and analysis of the model simulation results to attain better model simulation results.
2. Improve the spatial resolution of the model simulations and execute scale conversion. Point-scale observation information can be adopted as the basis for scale conversion to obtain the high-precision driving data required for regional remote sensing ET models. With the development and advancement of science and technology, more remote sensing data and reanalysis data with a high accuracy and long time series will be obtained, and the speed of computer operations will increase. Moreover, these data could be more widely applied to research in all walks of life and more value could be gained.
3. In the future, it is necessary to continue to analyse the temporal and spatial variabilities in other factors related to ET, examine the relationship between these factors, and analyse the driving elements of these factors.

## Data Availability

The datasets generated during and/or analysed during the current study are available in the github repository: https://github.com/zhangfeng0826/ETdata. The datasets has been used to generate the maps. The figures are generated by python code, the python code named ‘ET data process Python codes.zip’ has been uploaded in the github repository: https://github.com/zhangfeng0826/ETdata.
